# Bond Behavior of Plain Bars in Concrete under Reversed Cyclic Loading

**DOI:** 10.3390/ma16134836

**Published:** 2023-07-05

**Authors:** Jun Zhao, Lu Yin, Xiaopeng Li, Xinjie Yue

**Affiliations:** School of Civil Engineering, Zhengzhou University, Zhengzhou 450001, China; zhaoj@zzu.edu.cn (J.Z.);

**Keywords:** plain bar, monotonic load, reversed cyclic load, the maximum bond stress, bond stress–slip model

## Abstract

Plain bars with a diameter of 10 mm are widely used in reinforced concrete buildings, and the bond behavior between the bars and concrete has an essential effect on the seismic performance of concrete structures. Thus, to assess the safety of old buildings and repaired buildings with normal concrete, it was necessary to further investigate the bond performance of the plain bars in the concrete. The bonding tests under monotonic and reversed cyclic loading were carried out on the specimens reinforced with plain bars, and the influences of concrete grade and embedment length on the bond behavior were taken into consideration. The results indicate the maximum bond stress under reversed cyclic loading is less than that under monotonic loading, and this is the same for corresponding slip for the same test parameters. The concrete compressive strength positively affects the maximum bond stress, whereas the embedment length has a negative effect. Based on the elasticity analysis and test data fitting, the expressions of bond stress at characteristic points on the bond stress–slip curves were carried out. Consequently, the bond stress–slip model was established, which could be applied to calculate the bond stress–slip relationships under monotonic and reversed cyclic loading. By comparison between the test curves and proposed model, a good agreement is observed, which indicates that the proposed model can be used to predict the bond stress–slip curve of plain bars in concrete.

## 1. Introduction

Many concrete buildings exist that were widely reinforced with plain bars up to the 1970’s [1]. Due to the low strength of materials and lack of guidance of modern design standards, concrete buildings exist that were reinforced with plain bars did not satisfy the safety requirements of modern society. Besides, the buildings reinforced with plain bars also needed to be repaired and strengthened by new concrete, due to durability deterioration, seismic damage, and so on. Thus, it was necessary to assess the safety of the historical and existing buildings to formulate effective maintenance interventions. The bond behavior between the bars and concrete was an essential performance, which had significant effects on the mechanic performance of concrete structures [2,3]. The experimental results indicate the bond performance not only determines the anchorage length of the plain bars, but affects the bearing and displacement capacities of the concrete structures. Arani et al. [4] conducted quasi-static tests on four concrete columns, utilizing the plain bars as longitudinal bars. The results show that the flexural state is a secondary action after cyclic load due to the low bond strength of plain bars. Thus, the axial load eccentricity was reduced from base moments, whereas it increased for the columns reinforced with deformed bars. Except for the influences of bond performance on the mechanical performance of concrete structures, an accurate bond stress–slip (*τ*–*s*) model of the plain bars in concrete were required when conducting numerical analysis and theoretical calculation on the seismic performance of the old and repaired concrete buildings. Therefore, it was desirable to study the bond behavior of plain bars in concrete to further assess the mechanical performance of the old and repaired concrete buildings.

A great deal of experimental and theoretical investigations were performed on the bond behavior between the plain bars and concrete, which involved factors included concrete types, freezing-and-thawing, corrosions, anchoring end details, loading rates, lateral forces, and so on. Xiao et al. [5], Hossain [6], Li et al. [7], Wu et al. [8], Purnomo et al. [9], Jiang et al. [10], Faleschini et al. [11], and Deng et al. [12] experimentally conducted the experimental investigations on the bond behavior of the plain bars in recycled aggregate concrete (RAC), lightweight volcanic pumice concrete (LVPC), highly ductile fiber-reinforced concrete (HDFC), machine-made sand concrete (MSC), sand-coated polypropylene coarse aggregate concrete (SPCAC), self-compacting lightweight aggregate concrete (SLAC), electric arc furnace slag concrete (EAFSC), and engineered cementitious composites (ECC), respectively. The results show the bond failure modes of the bond specimens made by the above concrete are all pull-out failure of plain bars. In addition, the RAC replacement ratio of RAC [5] and compression strength of LVPC [6] had no obvious effect on the bond strength of plain bars in concrete, but the bond strength increased with the increasing strength of MSC [7], HDFC [8], SPCAC [9], SLAC [10], and ECC [12]. The EAFSC with large mass ratio of water to cement had high bond strength [11]. Shang et al. [13] presented the influences of freezing–thawing cycles on the bond behavior of plain bars in RAC, and proposed an expression to depict the relationship between bond strength and freezing–thawing cycles. Ma et al. [14] and Robuschi et al. [15] reported the corrosion effects on the bond behavior of plain bars in concrete, which showed the corrosion levels prominently impacted the *τ*–*s* relationships and bonding failure modes. Fabbrocino et al. [16] performed both beam and pull-out tests to study the influences of anchoring end details (including straight and hooked ends) of plain bars on the bond behavior. Zhang et al. [17], Wu et al. [18], Xu et al. [19], Li et al. [20], and Li et al. [21] systematically studied the bond performance of the plain bars in concrete under lateral force through several series of pull-out tests, with the combinations of lateral tensions, biaxial lateral tensile–compressive stresses, uniaxial and biaxial lateral pressures, and loading rates were analyzed as well. The results show the bond strength of plain bars in concrete increase with the decreasing lateral tensile stress and increasing lateral compression stress. The *τ*–*s* models were established in the literature [5,9,12,17,18,19,20,21,22,23,24] by numerical regression of test data based on BPE model [25]. Zhang et al. [26] deduced an analytical solution for predicting the bond strength of plain bars under uniform lateral tension. Though numerous bonding tests have been reported and the corresponding *τ*–*s* models were established for the specimens reinforced with plain bars, the loading form of tests were just monotonic loading in the mentioned literature. The slip value set in the previous studies were less than 10 mm, which lacked the investigations on the bond behavior under monotonic loading at a large slip value.

The bond behavior under monotonic loading was commonly employed to calculate the anchor length of reinforcement bars embedded in the ends of the concrete members. However, for the seismic response, the failure zones of the concrete members were mostly located at the plastic hinge areas where the maximum bending moment happened, rather than anchor positions. Under the earthquake action, the plastic hinge areas generally were subjected to the reversed cyclic loading, resulting in reversed cyclic bond response. Thus, to make the studies on the bond performance close to the actual seismic condition, some researchers conducted the bonding tests under cyclic loading. Pan et al. [27] reported the bond behavior of plain bars in highly ductile concrete under repeated loading, in which the loads was applied positively and unloaded to zero in each loading cycle and the bond behavior of PE fiber and PVA fiber-reinforced specimens was analyzed. Verderame et al. [28,29] conducted experimental and analytical investigations on the bond behavior of plain bar in low-strength concrete under cyclic loading, where plain bars with a diameter of 12 mm and a yield strength of 330 MPa and the concrete with a compression strength of 15.9 MPa were adopted. The peak slips in all loading cycles were the same. Xa et al. [30] experimentally investigated bond behavior of plain bars in frost-damaged concrete under cyclic loading, where the plain bar with a diameter of 16 mm and a yield strength of 263.3 MPa was employed. In addition to the plain bars, experiments were carried out to study the bond behavior of deformed bars [31,32] and FRP bars [33,34] in concrete under cyclic loading. It can be summarized that there is a lack of a sufficient study on the bond behavior of plain bars in normal concrete under reversed cyclic loading at present. To obtain the cyclic bond performance, it was necessary to conduct the bonding tests of the plain bars in normal concrete under reversed cyclic loading.

Except for the experimental investigations, several studies indicated that the *τ*–*s* model was very important for seismic analysis of concrete structures and members. Cai et al. [35], Sargsyan et al. [36], Grigor et al. [37], and Liu et al. [38] conducted seismic calculations on the concrete columns, which showed the calculated results were more accurate when the theoretical seismic calculation took the *τ*–*s* model into account. However, a suitable *τ*–*s* model of plain bars in normal concrete has not been established yet, which limits the theoretical and numerical analysis on repaired buildings strengthened by the normal strength concrete. Therefore, it was necessary to propose an accurate *τ*–*s* model that could be applied for the seismic calculation of a building reinforced with plain bars.

**Figure 1:**
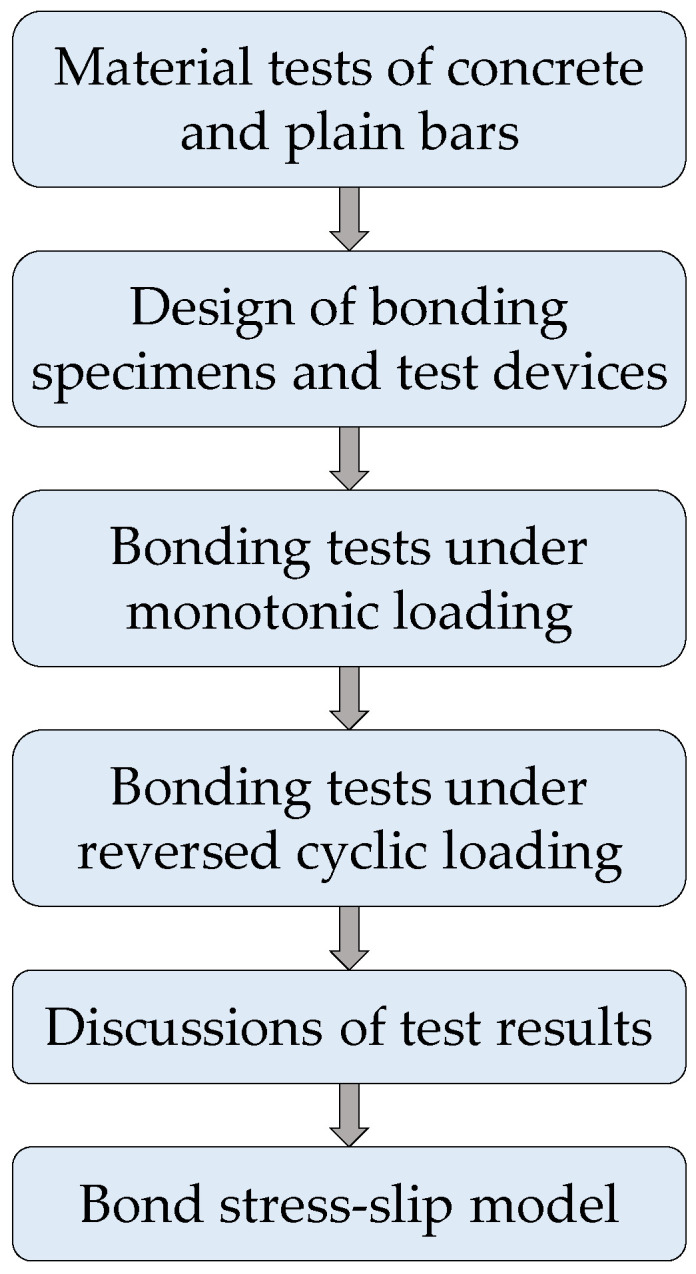
Flow chart of the research.

The main purpose of the study was to obtain the bond behavior of plain bars with a diameter of 10 mm in normal strength concrete under reversed cyclic loading, and propose a *τ*–*s* model of plain bars in normal concrete under reversed cyclic loading for conducting the seismic analysis of buildings reinforced with plain bars. The flow chart of the research is shown in Figure 1. The pull-out tests under both monotonic and reversed cyclic loading were performed, in which the concrete grade and embedment length were considered. To reveal the bond behavior under monotonic loading at a large slip value, the target value of tested slip in monotonic loading tests was set as 40 mm. Additionally, the bond stresses of feature points on the *τ*–*s* curves were carried out according to the theoretical analysis of elasticity and the numerical fitting of test results. Finally, a simplified *τ*–*s* model considering the bond strength degeneration was established, which was appropriate for describing the *τ*–*s* curves under reversed cyclic loading. 

## 2. Materials and Methods

### 2.1. Materials

The mix proportions of concrete are displayed in Table 1. Three kinds of strength grades were designed for concrete in accordance with Chinese specification JGJ 55-2011 [39]. The cement used P.O 42.5 Portland cement, natural river sand with a fineness modulus was 2.7, and the maximum size of coarse aggregate size was 20 mm. The cube compression strength *f*_cu_ was obtained by concrete compression test with three cubic samples of 150 mm × 150 mm × 150 mm according to Chinese standard GB/T 50081-2019 [40], as seen in Table 1.

**Figure 2:**
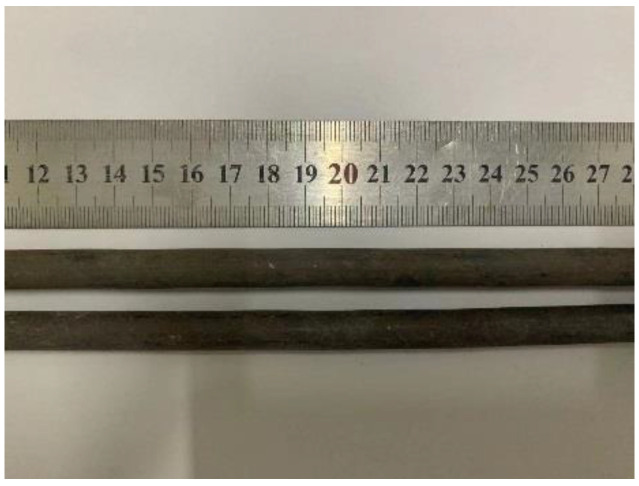
Photograph of the plain bars.

The plain bars used HPB335 steel bars with a diameter of 10 mm in the bonding tests, whose yield and tensile strength were 368 MPa and 529 MPa with SD and were 2.828 MPa and 3.859 MPa, respectively. The elongation was 28.75%. The mechanical properties were measured by static tensile tests on three samples according to Chinese standard GB/T 228.1-2021 [41]. The used plain bar is shown in Figure 2.

### 2.2. Bonding Specimens

**Figure 3:**
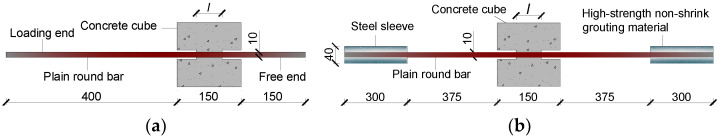
Design details of the bonding specimens: (**a**) monotonic specimen; (**b**) reversed cyclic specimen.

Figure 3a,b demonstrates the design details of bonding specimens under monotonic and reversed cyclic loading, respectively. Each specimen group had six bonding specimens, among which three were tested by monotonic tests and the other three were subjected to reversed cyclic tests. The six specimens in the same specimen group adopted identical concrete compressive strength and embedment length. The size of concrete cube in all bonding specimens was 150 mm × 150 mm × 150 mm. The embedment lengths were 50, 75, and 100 mm, which were 5, 7.5, and 10 times of the plain bar diameter, respectively. The overall length of plain bar in the monotonic specimen was 700 mm and that in the reversed cyclic specimen was 1500 mm. When casting the specimens, two pieces of polyvinyl chloride (PVC) pipe with a diameter of 12 mm were installed in the non-bonding sections of the plain bar to prevent cement grout from flowing into it. After the manufacture was completed, both ends of the plain bar were inserted into steel pipes for the reversed cyclic specimen, and then high-strength non-shrink grouting material was poured into. The tested parameters of each specimen group are presented in Table 2.

### 2.3. Test Devices and Procedures

**Figure 4:**
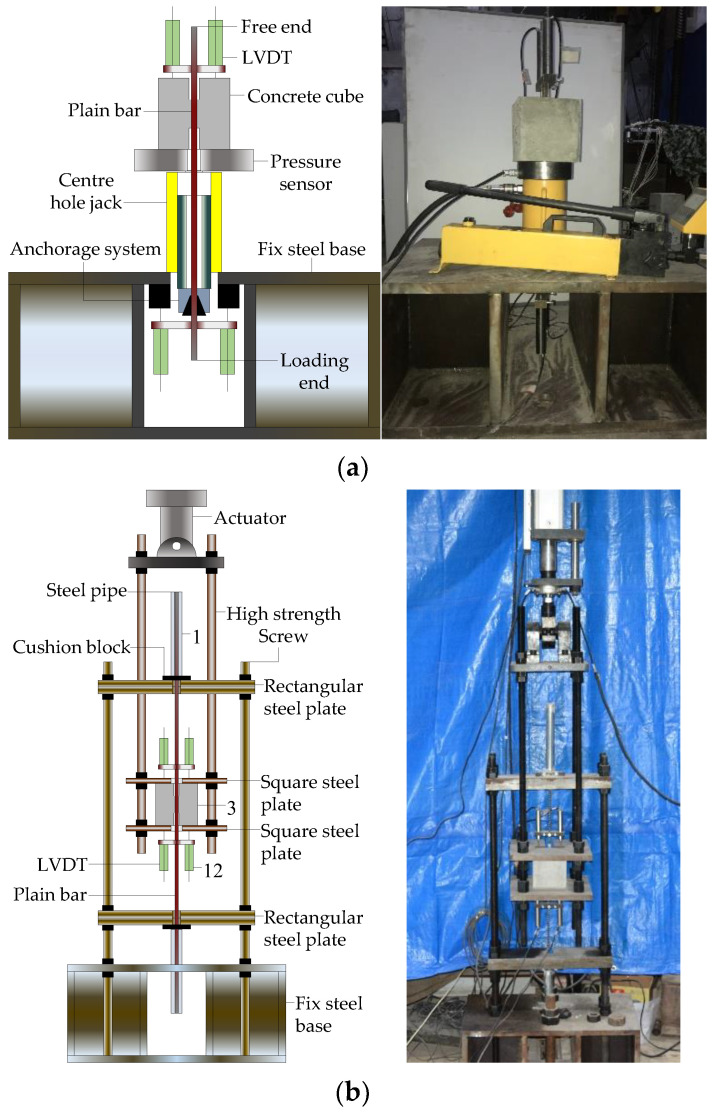
Schematic diagrams and photographs of test set-up: (**a**) monotonic loading; (**b**) reversed cyclic loading.

As shown in Figure 4a, a monotonic loading test device was designed and fabricated in accordance with ASTM D7913 [42], which consisted of a manual pump, a centre hole jack, a pressure sensor, a set of wedge-type anchors, and a fixed steel base. The wedge-type anchor was set on the loading end of plain bar. The manual pump provided the hydraulic pressure for the central-hole jack. Subsequently, the piston rod of the jack drove the wedge-type anchor, moving downward together with plain bar. The pressure sensor was placed between the jack and the concrete cube to measure the load values. Two linear variable differential transformers (LVDTs) were arranged at the free end of the plain bar to measure the slip values. The loading rate 0.02 mm/s was set in monotonic tests.

Figure 4b shows the reversed cyclic loading test device, which included an electrohydraulic servo system, a loading frame, a supporting frame, and a fixed steel base. The electrohydraulic servo system was employed to apply reversed cyclic loading. The loading frame was formed by two square steel plates and four high-strength screws. The supporting frame was formed by two rectangular steel plates and four high-strength screws. The loading frame and the supporting frame were tightly connected with the actuator of the electrohydraulic servo system and the fixed steel base, respectively. In the process of the test, the actuator applied the reversed cyclic loading on the loading frame, which drove the concrete cube of the specimen up and down.

**Figure 5:**
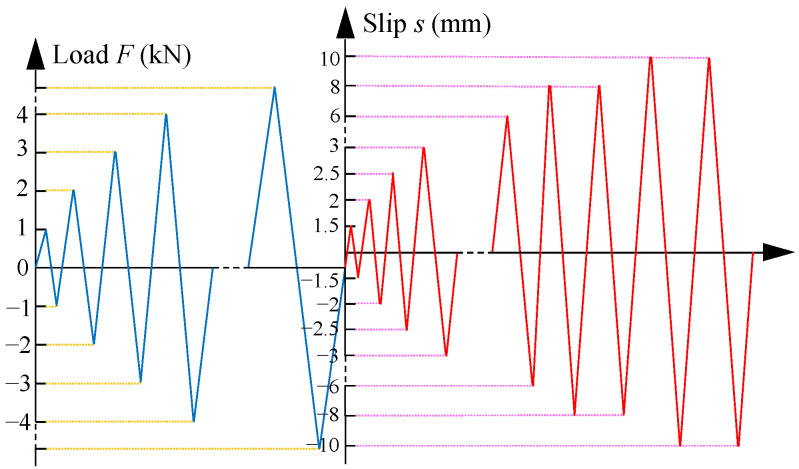
Loading scheme for reversed cyclic loading.

The loading scheme for the reversed cyclic loading tests is presented in Figure 5. When the slips were less than 1.5 mm, a single cycle was conducted in each loading level and the increments in peak loads in each loading cycle in the positive and negative directions were ±1 kN. When the peak slips of the loading cycle exceed 1.5 mm, a total of 13 loading cycles were conducted, with the peak slips of the loading cycles being ±1.5 mm, ±2 mm, ±2.5 mm, ±3 mm, ±3.5 mm, ±4 mm, ±4.5 mm, ±5 mm, ±6 mm, ±8 mm, ±8 mm, ±10 mm, and ±10 mm. The loading rate was 1.2 mm/min before the peak slip of ±4 mm and then increased to 2.0 mm/min after the peak slip of ±4 mm.

## 3. Results and Discussions

### 3.1. Failure Mode

**Figure 6:**
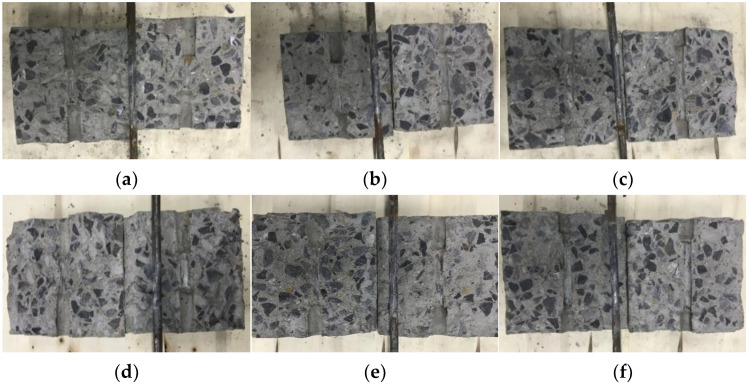
Failure state of bonding surface: (**a**–**c**) are C30-D10-50, C30-D10-75, and C30-D10-100 specimens under monotonic loading; (**d**–**f**) are C30-D10-50, C30-D10-75, and C30-D10-100 specimens under reversed cyclic loading.

All specimens present pull-out failure in the monotonic and reversed cyclic loading tests. In the process of the loading, the plain bar was pulled out from the concrete cube. Simultaneously, no concrete splitting failure happened and no crack appeared on the surface of the concrete cube. After the bonding tests, the specimens split, and the bonding surface of the plain bar and concrete are presented in Figure 6. As seen in Figure 6, the specimens under monotonic and reversed cyclic loading have a similar failure state of the bonding surface. The plain bars present steel polish because of the abrasion wear of the surface layer. The bonding surface of concrete is also scuffed, and produces some concrete powder.

### 3.2. Bond Stress–Slip Curves

The applied force *F* and the corresponding slip *s* of plain bar relative to the concrete was measured over the course of the test. The local bond stress *τ* was calculated as the average along the embedment length of the plain bar, as follows:

(1) τ=F/(πdl)

The test results of the main parameters in the monotonic and reversed cyclic loading tests are presented in Table 3. For monotonic loading tests, $\tau_{e}^{m}$ is the peak bond stress of the elastic stage; $\tau_{a}^{m}$ is the bond stress at abrupt point, and $s_{a}^{m}$ is the slip corresponding to $\tau_{a}^{m}$; $\tau_{u}^{m}$ and $\tau_{r}^{m}$ are the maximum bond stress and the residual bond stress, respectively. For reversed cyclic loading tests, $\tau_{u}^{+}$ and $\tau_{u}^{-}$ are the maximum bond stresses in the positive and negative directions, respectively. $\tau_{10,1}^{+}$ and $\tau_{10,1}^{-}$ are the bond stresses at the slip of 10 mm and −10 mm in the first cycle of ±10 mm, respectively. *φ* is the degradation coefficient of the maximum bond stress in the negative direction to that in the positive direction for one specimen, which is $\varphi =\tau_{u}^{-}/\tau_{u}^{+}$. *ζ* is the degradation coefficient of the maximum bond stress under reversed cyclic loading to that under monotonic loading for the same specimen group, which is $\zeta =\tau_{u}^{+}/\tau_{u}^{m}$, where $\tau_{u}^{m}$ and $\tau_{u}^{+}$ adopt the mean values of the same specimen group in Table 3.

#### 3.2.1. Bond Stress–Slip Curves under Monotonic Loading

**Figure 7:**
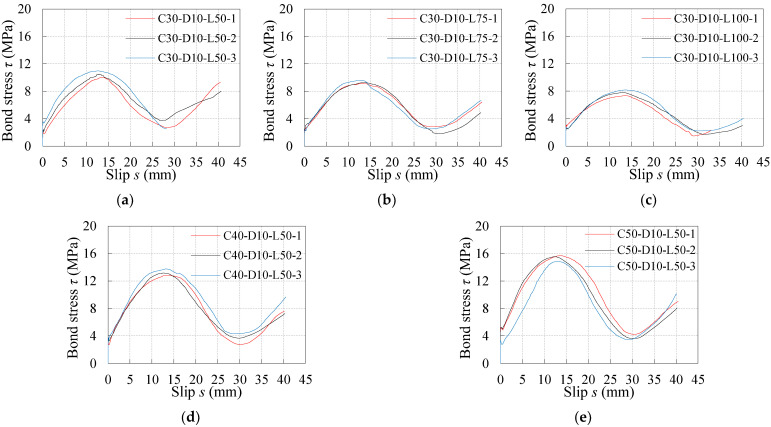
Bond stress–slip curves under monotonic loading: (**a**) C30-D10-L50; (**b**) C30-D10-L75; (**c**) C30-D10-L100; (**d**) C40-D10-L50; (**e**) C50-D10-L50.

**Figure 8:**
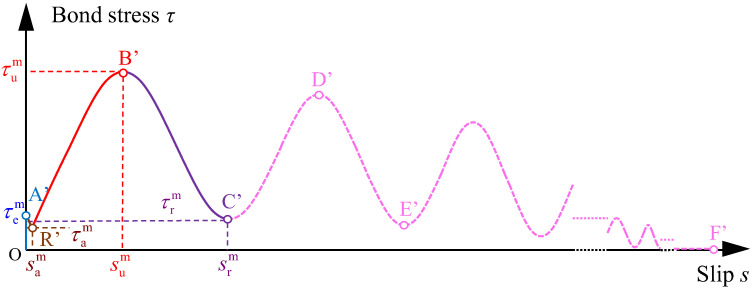
Schematic diagram of bond stress–slip curve under monotonic loading.

Figure 7 records the *τ*–*s* curves under monotonic loading for all three samples in each specimen group. As seen from Figure 7, the shapes of *τ*–*s* curves under monotonic loading for the specimens with different concrete compression strengths and embedment lengths are similar. On the basis of curve characters, the schematic diagram of the *τ*–*s* curve under monotonic loading is drawn in Figure 8. The *τ*–*s* curve under monotonic loading is divided into five stages, the characteristics of which are observed and summarized as follows:

Elastic stage (OA’ segment): the bond stress increases with little increasing slip at the stage, thus, the slope of the OA’ segment tends to infinity and the slip of point A could be regarded as 0 mm. The chemical adhesion provides the mainly bond resistance. At the point A’ ($0,\;\tau_{e}^{m}$), the bond stress reaches the peak bond stress $\tau_{e}^{m}$ at the elastic stage.

Abrupt stage (A’R’ segment): the chemical adhesion fails after the point A’ ($0,\;\tau_{e}^{m}$) as the slip occurs. Simultaneously, the bond stress appears at a sudden drop and the slip increases abruptly. Then the curve suddenly develops to the point R’ ($s_{a}^{m},\;\tau_{a}^{m}$), which is a characteristic point named ‘abrupt point’ in the study. It can be learnt from Table 3 that the bond stress $\tau_{a}^{m}$ us less 0.16–0.35 MPa relative to $\tau_{e}^{m}$, and the corresponding slip $s_{a}^{m}$ basically ranges in 0.20–0.36 mm for all the specimens.

Strengthening stage (R’B’ segment): after the abrupt point R’ ($s_{a}^{m},\;\tau_{a}^{m}$), the bond stress increases nonlinearly and slowly as the slip increases until to the maximum bond of the R’B’ segment decreases sharply, which indicates the bond stiffness. The bond resistance is mainly offered by sliding friction in the stage. For all specimens, the slip $s_{u}^{m}$ is in the range of 12.54–13.62 mm. The consistent growth of bond stress after the point R’ is caused by the following reasons: after the point R’, the interfacial surface of concrete has not been fully destroyed, and the surface of plain bar is still rough. Therefore, the sliding friction still exists between the plain bar and concrete under the external loading, and continues to provide the bond resistance. 

Degradation stage (B’C’ segment): after the point B’ ($s_{u}^{m},\;\tau_{u}^{m}$), the bond stress gradually decreases with the increasing slip. The friction becomes lower and lower because the bonding surface is gradually damaged. The bond stress reaches the minimum of the B’C’ segment at the point C’ ($s_{r}^{m},\;\tau_{r}^{m}$). The bond stress of the point C’ is defined as the residual bond stress $\tau_{r}^{m}$ under monotonic loading in the study. For all specimens, the degeneration factor *η* and the slip $s_{r}^{m}$ are in the range of 0.20–0.36 and 26.73–31.27 mm, respectively. It can be observed the R’B’ segment and the B’C’ segment are roughly symmetrical about the vertical line $s=s_{u}^{m}$. In addition, the shape of the R’B’C’ segment presents as half a period sine wave.

Attenuation stage (C’F’ segment): The C’F’ segment was predicted on the basis of the test results. After the point C’ ($s_{r}^{m},\;\tau_{r}^{m}$), the bond stress grows again with the increase in slip, and then reaches the peak value once again, which is the point D’. This test phenomenon is due to the design of the specimens. Though the initial embedment segment of the plain bar was pulled out from the concrete, the non-bonding segment of free end was pulled into and came into contact with the concrete cube at the same time. Therefore, the new segment of the plain bar enhances the friction due to rough surface, resulting in the increase in bond stress. The bond stress at the point D’ is lower than that at the point B’ due to the damage of the bonding surface. After the point D’, the bond stress decreases to the next valley point E’ due to the further damage of the bonding surface. The shape of the attenuation stage is similar as a decaying sine wave, whose bond stresses of peak and valley value declines until it is close to 0 MPa.

#### 3.2.2. Bond Stress–Slip Curves under Reversed Cyclic Loading

**Figure 9:**
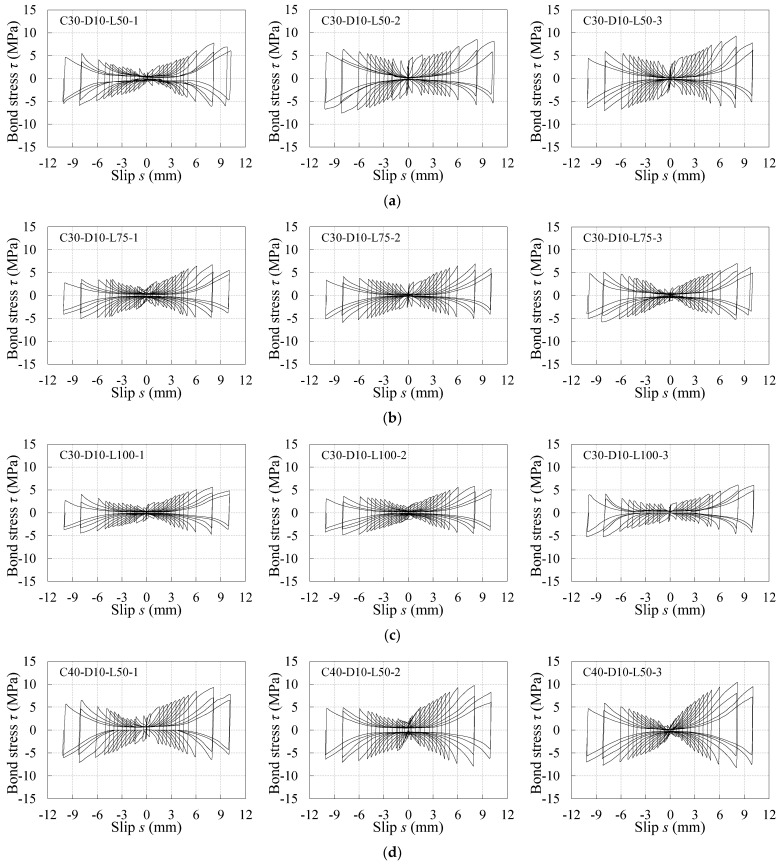

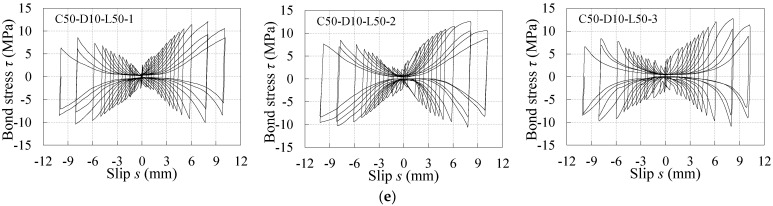
Bond stress–slip curves under reversed cyclic loading: (**a**) C30-D10-L50; (**b**) C30-D10-L75; (**c**) C30-D10-L100; (**d**) C40-D10-L50; (**e**) C50-D10-L50.

**Figure 10:**
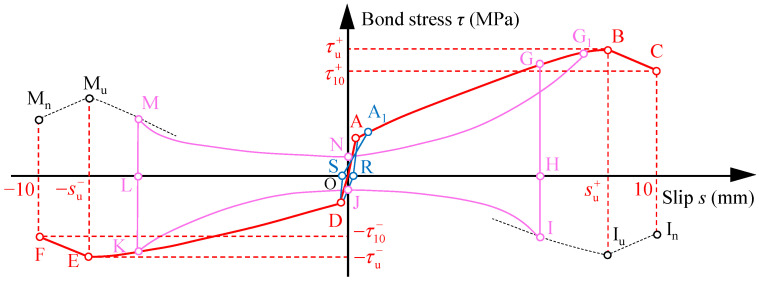
Schematic diagram of bond stress–slip curve under reversed cyclic loading.

The *τ*–*s* curves under reversed cyclic loading for all specimens are reported in Figure 9 in term of the test results, which present as the butterfly shape and is roughly symmetrical about the origin. The shapes of *τ*–*s* curves under reversed cyclic loading for the specimens with different concrete compression strengths and embedment lengths are similar. The schematic diagram of the *τ*–*s* curve under reversed cyclic loading is summarized in Figure 10, where the curve FEDOABC represents the skeleton curve. Based on the test curves, the development of the *τ*–*s* curve under reversed cyclic loading is divided into three stages. As the laws of the *τ*–*s* curve in the positive and negative directions are the same, only the first-half skeleton curve and hysteresis loop are analyzed, as follows:

(1) Elastic stage (OA and OD segments): The bond stress basically increases with the increasing slip at the stage. The point A represents the ending point of the elastic stage, whose bond stress and slip are very small. The chemical adhesion and friction mainly provide bond resistance at the stage. Once the slip occurs, the chemical adhesion fails as well. The hysteresis loop in this stage is similar to the curve ARDSA_1_. As unloaded to 0 kN at the point R, the slip of the point R cannot return to 0 mm, but is larger than the slip of the unloading point A. Then the curve directly reaches the next unloading point D in the negative direction;

(2) Strengthening stage (AB and DE segments): As the loading level increases, the peak bond stress $\tau_{u,i}^{+}$ of each cycle increases nonlinearly until reaching the maximum bond stress $\tau_{u}^{+}$ at the point B ($s_{u}^{+},\;\tau_{u}^{+}$). The slopes gradually decrease in the AB segment, which indicates the bonding stiffness becomes weaker and weaker. It is because the bonding surface of the concrete is gradually damaged and the plain bar is worn. The bond resistance is mainly provided by the sliding friction at the stage.

For all specimens, the maximum bond stresses $\tau_{u}^{+}$ and $\tau_{u}^{-}$ generally happen in the first loading cycle of ±8 mm, thus, the corresponding slips $s_{u}^{+}$ and $s_{u}^{-}$ are considered to be equal to 8 mm. $\tau_{u}^{-}$ is always less than $\tau_{u}^{+}$ for the same specimen, the maximum degradation is 28%, as seen the degradation coefficient *φ* listed in Table 3. In addition, the mean test value of $\tau_{u}^{+}$ is less than that of $\tau_{u}^{m}$ for the same specimen group, whose degradation coefficient *ζ* ranges between 0.72–0.81, which indicates the bond strength degrades due to the action of the reversed cyclic loading. The slips $s_{u}^{+}$ are also less than $s_{u}^{m}$, which indicates the bond stiffness before the maximum bond stress degrades when the specimen suffers the reversed cyclic loading;

(3) Degradation stage (BC and EF segments): After the maximum bond stress $\tau_{u}^{+}$, the peak bond stress of the loading cycle of ±10 mm appears to decrease, which is caused by the abrasion on the bonding surface of both the plain bar and concrete. 

The typical hysteresis loop in both strengthening stage and degradation stage is similar to curve GHIJKLMNG_1_, as shown in Figure 10, a half of which was discussed as follows:

GH segment: This segment is the unloading stage. The point G is any point on the ABC segment, which represents the peak point of loading level. At the point H, the loading unloads to 0 kN. As the friction between the plain bar and concrete limits the restoration of slip, the slips at point G and point H are almost equal to each other.

HI segment: This segment is the static frictional stage. After point I, the loading begins to act negatively. In the segment, the applied force is always less than the static friction between the plain bar and concrete until point I, resulting in few changes in slip in the HI segment. Thus, the slip of point I was assumed to be equal to that of the point G. The bond stresses at point I and point M are signed as $-\tau_{f}^{-}$ and $\tau_{f}^{+}$, whose envelop curves are curve II_u_I_n_ and MM_u_M_n_, respectively. As seen from the test results, $\tau_{f}^{-}$ and $\tau_{f}^{+}$ increase with the increase in $\tau_{u,i}^{+}$ and $\tau_{u,i}^{-}$, respectively. 

IJ segment: At point I, the slip begins to obviously change as the applied force is larger than the static friction. However, the bond stress decreases with the change in slip. The minimum bond stress $\tau_{f}^{-}$ appears until the curve reaches point J, whose slip is about 0 mm. For some samples of the specimen group C30-D10-L100, C40-D10-L50, and C50-D10-L50, $\tau_{f}^{-}$ even decreases to 0 MPa after the loading cycle of ±1.5 mm.

JK segment: After point J, the bond stress increases again with the change in slip until the curve reaches the negative peak point K.

### 3.3. The Influence of Test Parameters on the Bond Performance

It can be learnt from Table 3 and Figure 7 and Figure 9 that the influence laws of concrete compression strength and embedment length on the maximum bond stress under reversed cyclic loading show a similar tendency to that under monotonic loading. 

For the specimens with given embedment length, the concrete compression strength has a positive effect on the maximum bond stress. The maximum bond stress increases with the increase in concrete compressive strength. For example, for the reversed cyclic specimens with an embedment length of 50 mm, the maximum bond stress of C40 and C50 specimens increase by 15.4% and 47.3% relative to that of C30 specimens, while it increases by 11.5% and 29.6% for the monotonic specimens. Based on the above change laws, it could be deduced that the bond strength of the plain bars in the old concrete is lower than the normal concrete due to the low strength and durability degradation of the old concrete.

For the specimens with given concrete compression strength, the maximum bond stress decreases with the increasing embedment length. For example, for the reversed cyclic specimens with C30 concrete, the maximum bond stress of the specimens with embedment lengths of 75 mm and 100 mm decreases by 15.4% and 31.3% relative to that of the specimens with embedment lengths of 50 mm, while it decreases by 21.2% and 39.1% for the monotonic specimens.

In addition, there is no obvious influence on the slip corresponding to the maximum bond stress for the specimens under either reversed cyclic or monotonic loading.

## 4. Analytical Model of Bond Stress-Slip Curve

**Figure 11:**
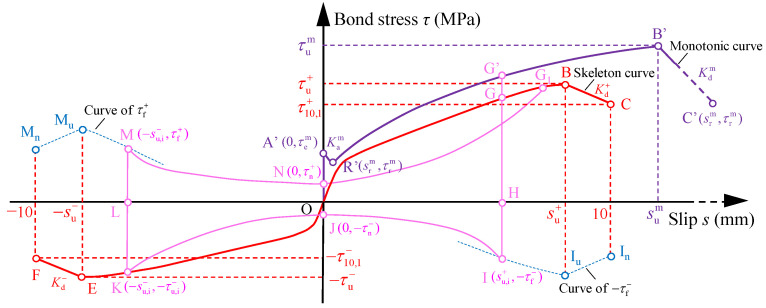
Schematic diagram of bond stress–slip model.

To apply the *τ*–*s* relationship of plain bars in concrete to the seismic analyses of existing concrete building, it was necessary to propose a mathematical model of the *τ*–*s* relationship. According to the observations and discussions on the test curves in Section 3.2, the basic rules of the *τ*–*s* model are schematically illustrated in Figure 11 and described as follows:

For the monotonic curve OA’R’B’C’ in the *τ*–*s* model, the OA’, A’R’, R’B’, and B’C’ segment represent the elastic stage, abrupt stage, strengthening stage, and degradation stage, respectively. In the model, assuming the slip of point A ($s_{e}^{m},\;\tau_{e}^{m}$) is equal to 0 mm, which is $s_{e}^{m}=0$. The abrupt stage and degradation stage (B’C’ segment) are assumed as two straight lines with the slopes of $K_{a}^{m}$ and $K_{d}^{m}$, respectively. 

For the *τ*–*s* model under reversed cyclic loading, the skeleton curve could determine the peak point of each loading level. Both positive and negative skeleton curve FEOBC were simplified as two stages, including ascent and degradation stages. The ascent stage (OB and OE segments) consists of the elastic stage and strengthening stage mentioned in Section 3.2.2. The degradation stage (BC and EF segments) is simplified as two straight lines with the slopes of $K_{d}^{+}$ and $K_{d}^{-}$, respectively. The curve G’GHIJKLMNG_1_ represents one typical hysteresis loop in the model, with rules stipulated as follows: Before the first unloading point G’, the *τ*–*s* curve traces along the monotonic curve OA’R’B’C’. The point G’ is any point of the monotonic curve. Then the unloading branch (G’H segment) forms, which intersects with the skeleton curve OBC at the point G ($s_{u,i}^{+},\;\tau_{u,i}^{+}$) and ends at the point H ($s_{u,i}^{+},\;0$). Subsequently, the static frictional branch (HI segment) is followed from point H to I in the negative direction. Due to few changes in slip within the segment G’I, the slope of segment G’I is assumed as infinity. The curve II_u_I_n_ represents the envelop curve of the point I ($s_{u,i}^{+},-\tau_{f}^{-}$) in a different loading cycle. After point I, the decrease branch (IJ segment) develops to point J, where the minimum bond stress of IJ segment happens. The slip of point J is approximate to 0 mm and the point J is marked as ($0,-\tau_{n}^{-}$) in the model. Then the re-increase branch (JK segment) is traced from point J to the unloading point K ($-s_{u,i}^{-},-\tau_{u,i}^{-}$) in the negative direction. The segment IJK is regarded as a quadratic parabola in the model according to the shape of the test curves. After point K, the other half hysteresis loop LMNG_1_ experiences the unloading branch (KL segment), static frictional branch (LM segment), decrease branch (MN segment), and re-increase branch (NG1 segment), whose rules are the same as the curve GHIJK. In addition, the coordinates of characteristic points M and N are marked as ($-s_{u,i}^{-},\;\tau_{f}^{+}$) and ($0,\;\tau_{n}^{+}$). At the next unloading point G_1_ in the skeleton curve, the completed hysteresis loop GHIJKLMNG1 is completed.

According to the rules mentioned above, the *τ*–*s* model between the plain bar and concrete was needed to be expressed by the following functions: (1) the formulae of the monotonic curve and skeleton curve; (2) the expressions of each segment of the hysteresis loop; (3) the expressions of bond stress of the characteristic points.

### 4.1. Elastic Analysis on Bond Stress 

**Figure 12:**
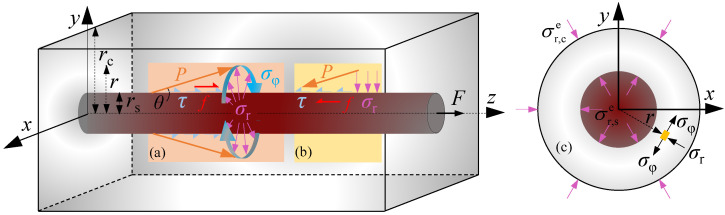
Stress state in elastic stage: (**a**) surround concrete; (**b**) plain bar; (**c**) thick-walled cylinder model.

Figure 12a,b show the stress state that took the surround concrete and the plain bar as study objects, respectively. In Figure 12, *F* is the applied force, *f* is friction, *τ* is bond stress, *σ*_r_ and *σ*_φ_ are radial stress and hoop stress of the analysis infinitesimal element, respectively. *r*_s_ and *r*_c_ are the radius of the plain bar and half of the side length of concrete cube, respectively. As the slip occurs, interaction force *P* between the plain bar and concrete is generated along the diagonal direction with a *θ* degree. The interaction force *P* could be decomposed into the bond stress *τ* and the radial stress *σ*_r_.

According to the bond mechanism between the plain bar and concrete, the bond stress *τ* was mainly provided by the friction *f*, thus:

(2) τ=f

Based on the tribological principle [43], the relationship between the friction *f* and the radial stress *σ*_r_ was obtained as:

(3) $$f=\mu \sigma_{r}$$

where *μ* is the friction coefficient, which was taken as 0.35 [44] in the study.

Thus,

(4) $$\tau =\mu \sigma_{r}$$

Based on the thick-walled cylinder model proposed in elasticity [45], the bonding specimen was regarded as a thick-walled cylinder, as seen in Figure 12c. Figure 12c presents the stress state of the infinitesimal element at a distance of *r* from the center of the cross-section of the plain bar. The inner and outer rings are the plain bar and the inscribed cylinder of the concrete cube, whose radius is marked as *r*_s_ and *r*_c_, respectively. The internal radial stress $\sigma_{r,s}^{e}$ is produced by the friction, and the outer radial stress is equal to 0 MPa. Therefore, the stress boundary conditions of the thick-walled cylinder were written as:

(5) $${\sigma_{r}|}_{r=r_{s}}=-\sigma_{r,s}^{e};{\;\sigma_{r}|}_{r=r_{c}}=0$$

According to the elasticity, the radial stress *σ*_r_ and the hoop stress *σ*_φ_ of the analysis infinitesimal element at any point with the radius *r* could be deduced as:

(6) $$\left\{ \begin{array}{l} \sigma_{r}=-\sigma_{r,s}^{e}\frac{r_{s}^{2}}{r_{c}^{2}-r_{s}^{2}}\left(1-\frac{r_{c}^{2}}{r^{2}}\right)\\ \sigma_{\varphi }=\sigma_{r,s}^{e}\frac{r_{s}^{2}}{r_{c}^{2}-r_{s}^{2}}\left(1+\frac{r_{c}^{2}}{r^{2}}\right) \end{array}\right.$$

At the end of the elastic stage, the microcracks appear on the interfacial surface of concrete. Assuming the hoop stress *σ*_φ_ is equal to concrete tensile strength, which is:

(7) $$\sigma_{\varphi }=f_{t}$$

where the formula of tensile strength adopted that recommended in Chinese code [46] as:

(8) $$f_{t}=0.88\times 0.395f_{\mathrm{cu}}^{0.55}$$

Combining Equations (5)–(8), the radial stress *σ*_r_ is given as

(9) $$\sigma_{r}=f_{t}\frac{r_{c}^{2}-r^{2}}{r_{c}^{2}+r^{2}}$$

Considering the bonding failure happens on the bonding surface, thus, *r* = *r*_s_ is adopted when calculating the radial stress *σ*_r_. Further, the bond stress *τ* is expressed as:

(10) $$\tau =\mu f_{t}\frac{r_{c}^{2}-r_{s}^{2}}{r_{c}^{2}+r_{s}^{2}}$$

### 4.2. Monotonic and Skeleton Curves

#### 4.2.1. Elastic Stage of Monotonic Curve

For the elastic stage (OA’ segment) of monotonic curve, the point A’ ($0,\;\tau_{e}^{m}$) is the ending point and OA’ segment is a vertical curve, as shown in Figure 11. The peak bond stress $\tau_{e}^{m}$ in the elastic stage was proposed as Equation (11) by fitting test results based on Equation (10), which is written as:

(11) $$\tau_{e}^{m}=\frac{2.7}{\psi }\mu f_{t}\frac{r_{c}^{2}-r_{s}^{2}}{r_{c}^{2}+r_{s}^{2}}$$

where, ψ=0.1l/d+0.5 is the correction factor to consider the influence of the embedment length *l* and diameter *d* on the bond stress [47].

The tested and calculation results of $\tau_{e}^{m}$ are listed in Table 4, and the errors between them are within 7%.

Thus, the formula of elastic stage of monotonic curve is expressed as:

(12) $$\tau =\tau_{e}^{m};\;s=0$$

#### 4.2.2. Abrupt Stage of Monotonic Curve

For the abrupt stage (A’R’ segment) of monotonic curve, the points A’ ($0,\;\tau_{e}^{m}$) and R’ ($s_{a}^{m},\;\tau_{a}^{m}$) are the endpoints and A’R’ segment is a straight line with the slope of $K_{a}^{m}$. As shown in Table 3, it can be learned that the difference $\Delta =\tau_{e}^{m}-\tau_{a}^{m}$ and the slip $s_{a}^{m}$ are in the range of 0.16–0.35 MPa and 0.20–0.36 mm, respectively, which all display small discreteness. The average values 0.247 MPa and 0.269 mm were adopted for Δ and $s_{a}^{m}$ when calculating $\tau_{a}^{m}$ and $K_{a}^{m}$.

The expression of $\tau_{a}^{m}$ is given as:

(13) $$\tau_{a}^{m}=\tau_{e}^{m}-0.247$$

The slope of $K_{a}^{m}$ can be calculated by:

(14) $$K_{a}^{m}=\left(\tau_{a}^{m}-\tau_{e}^{m}\right)/s_{a}^{m}$$

According to two-point form of straight-line equation, the formula of the abrupt stage of the monotonic curve is expressed as:

(15) $$\tau =-0.918s+\tau_{a}^{m};\;0\leq s\leq s_{a}^{m}$$

#### 4.2.3. Strengthening Stage of Monotonic Curve and Ascent Stage of Skeleton Curve

In the strengthening stage of the monotonic curve and ascent branch of the skeleton curve, the maximum bond stresses are the key data for the *τ*–*s* model. As seen in Figure 11, the point B’ ($s_{u}^{m},\;\tau_{u}^{m}$) is the maximum bond stress point on the monotonic curve, and the point B ($s_{u}^{+},\;\tau_{u}^{+}$) and point E ($-s_{u}^{-},-\tau_{u}^{-}$) are that on the skeleton curve in the positive and negative directions, respectively.

For the maximum bond stress under monotonic loading, as seen from Table 3, the test results of ratio $\lambda =\tau_{e}^{m}/\tau_{u}^{m}$ are between 0.14–0.27 with a small standard deviation 0.035. The average value *λ* = 0.202 was adopted in the expression of $\tau_{u}^{m}$ as:

(16) $$\tau_{u}^{m}=4.95\tau_{e}^{m}$$

For the maximum bond stress under reversed cyclic loading, the degradation coefficients $\varphi =\tau_{u}^{-}/\tau_{u}^{+}$ and $\zeta =\tau_{u}^{+}/\tau_{u}^{m}$ are basically in the range of 0.76–0.90 and 0.72–0.81, respectively. The average value *φ* = 0.800 and *ζ* = 0.763 were used to calculate $\tau_{u}^{+}$ and $\tau_{u}^{-}$:

(17) $$\left\{ \begin{array}{l} \tau_{u}^{+}=0.763\tau_{u}^{m}\\ \tau_{u}^{-}=0.8\tau_{u}^{+} \end{array}\right.$$

The strengthening stage of the monotonic curve and the ascent stage of the skeleton curve can be established based on the BPE model reported by Eligehausen et al. [25], which is written as:

(18) $$\left\{ \begin{array}{l} \tau =\tau_{u}^{m}{\left(s/s_{u}^{m}\right)}^{\alpha^{m}};s_{a}^{m}\leq s\leq s_{u}^{m}\\ \tau =\tau_{u}^{+}{\left(s/s_{u}^{+}\right)}^{\alpha^{+}};\;0\leq s\leq s_{u}^{+}\\ \tau =-\tau_{u}^{-}{\left(-s/s_{u}^{-}\right)}^{\alpha^{-}};-s_{u}^{+}\leq s\leq 0 \end{array}\right.$$

As seen in Table 3, the test results of $s_{u}^{m}$ range from 12.54–13.62 mm for all specimens, which presents less dispersion. Thus, $s_{u}^{m}$ adopted the average value 13.12 mm in Equation (18). $s_{u}^{+}$ and $s_{u}^{-}$ are taken as 8 mm. *α*^m^, *α*^+^, and *α*^−^ are the shape parameters determined by the test curves, with test values listed in Table 3. The test values of *α*^m^, *α*^+^, and *α*^−^ basically change little for the specimens with different kinds of concrete compression strength and embedment length. Thus, when calculation was conducted on the *τ*–*s* curve, the average values of *α*^m^, *α*^+^, and *α*^−^ could be used in Equation (18), which are 0.377, 0.459, and 0.397, respectively. 

#### 4.2.4. Degradation Stages of Monotonic and Skeleton Curves

The degradation stages of the monotonic and skeleton curves were simplified as the straight lines B’C’, BC, and EF segments in Figure 11, whose slopes are marked as $K_{d}^{m}$, $K_{d}^{+}$, and $K_{d}^{-}$, respectively. $K_{d}^{m}$, $K_{d}^{+}$, and $K_{d}^{-}$ could be calculated by the endpoints of the B’C’, BC, and EF segments

(19) $$\left\{ \begin{array}{l} K_{d}^{m}=\left(\tau_{u}^{m}-\tau_{r}^{m}\right)/\left(s_{u}^{m}-s_{r}^{m}\right)\\ K_{d}^{+}=\left(\tau_{u}^{+}-\tau_{10,1}^{+}\right)/\left(s_{u}^{+}-10\right)\\ K_{d}^{-}=\left(-\tau_{u}^{-}+\tau_{10,1}^{-}\right)/\left(-s_{u}^{-}+10\right) \end{array}\right.$$

where $s_{u}^{m}=13.12$ and $s_{u}^{+}=s_{u}^{-}=8$ were adopted. The average value 29.61 mm was adopted for $s_{r}^{m}$ according to the test values presented in Table 3. The average values of test results $\tau_{r}^{m}/\tau_{u}^{m}$, $\tau_{10,1}^{+}/\tau_{u}^{+}$, and $\tau_{10,1}^{-}/\tau_{u}^{-}$ are 0.258, 0.872, and 0.880, respectively. Thus, the formulae of $\tau_{r}^{m}$, $\tau_{10,1}^{+}$, and $\tau_{10,1}^{-}$ could be expressed as:

(20) $$\left\{ \begin{array}{l} \tau_{r}^{m}=0.258\tau_{u}^{m}\\ \tau_{10,1}^{+}=0.872\tau_{u}^{+}\\ \tau_{10,1}^{-}=0.88\tau_{u}^{-} \end{array}\right.$$

Utilizing the two-point form of the straight-line equation, the degradation stages of monotonic and skeleton curves are given as:

(21) $$\left\{ \begin{array}{l} \tau =K_{d}^{m}\left(s-s_{u}^{m}\right)+\tau_{u}^{m};s>s_{u}^{m}\\ \tau =K_{d}^{+}\left(s-s_{u}^{+}\right)+\tau_{u}^{+};s>s_{u}^{+}\\ \tau =K_{d}^{+}\left(s+s_{u}^{-}\right)-\tau_{u}^{-};s<-s_{u}^{-} \end{array}\right.$$

Up to now, the formulae of monotonic curve and skeleton curve in the *τ*–*s* model have been obtained. As the slip is so large when the monotonic curve enters the attenuation stage, thus, the model before the slip of $s_{r}^{m}$ was proposed to predict the monotonic curve.

### 4.3. Hysteresis Loop

#### 4.3.1. Bond Stresses $\tau_{f}^{+}$ and $\tau_{f}^{-}$

Taking the embedment segment of plain bars in the unloading branch and static frictional branch (i.e., GI segment) as the studied object. Some assumptions were made based on the test results as follows: (1) no slip occurred in the GI segment; (2) no interface energy was lost for the plain bar in the GI segment; (3) no radial and hoop deformation were produced in the GI segment; (4) the decrease in the strain energy was caused by friction.

The elastic strain energy of the plain bar at point G and point I are written as Equations (22) and (23), respectively:

(22) $$U_{u,i}=\frac{1}{2}{∭}_{V}\sigma_{u,i}\varepsilon_{u,i}\mathrm{dV}$$

(23) $$U_{f}=\frac{1}{2}{∭}_{V}\sigma_{f}\varepsilon_{f}\mathrm{dV}$$

where *σ*_u,i_ and *ε*_u,i_ are the normal stress and corresponding normal strain at the point G; *σ*_f_ and *ε*_f_ are the normal stress and corresponding normal strain at point I.

The axial deformation Δ*l* of the plain bar was regarded as the displacement of the friction, which was calculated by:

(24) $$\Delta l=\varepsilon_{u,i}l$$

The friction *f*_s_ is equal to the bond resistance, which is

(25) $$f_{s}=\pi dl\tau$$

where *τ* adopted $\tau_{e}^{m}$ when calculation was performed.

The relationship between the normal stress and normal strain at the points G and I is written as:

(26) $$\left\{ \begin{array}{l} \sigma_{u,i}=E_{s}\varepsilon_{u,i}\\ \sigma_{f}=E_{s}\varepsilon_{f} \end{array}\right.$$

The relationship between the normal stress *σ* and local average bond stress *τ* is:

(27) σ=4τl/d

As the loading rates at point G and I are the same, the kinetic energy is unchanged at point I relative to that at point G. According to the law of energy conservation:

(28) $$U_{u,i}=\frac{1}{2}f_{s}\Delta l+U_{f}$$

Combining Equations (21)–(27), the expressions of $\tau_{f}^{+}$ and $\tau_{f}^{-}$ were obtained:

(29) $$\left\{ \begin{array}{l} \tau_{f}^{+}=\sqrt{\tau_{u,i}^{-}\left(\tau_{u,i}^{-}-\tau_{e}^{m}\right)}\;\\ \tau_{f}^{-}=\sqrt{\tau_{u,i}^{+}\left(\tau_{u,i}^{+}-\tau_{e}^{m}\right)} \end{array}\right.$$

Equation (28) is also the expression of bond stress of II_u_I_n_ and MM_u_M_n_.

**Figure 13:**
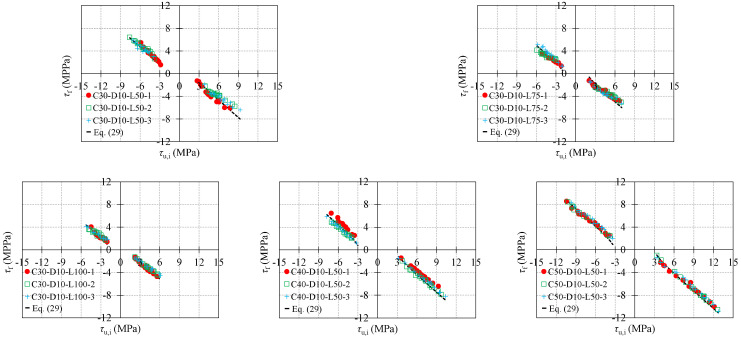
Relationship between $\tau_{f}$ and $\tau_{u,i}$.

**Figure 14:**
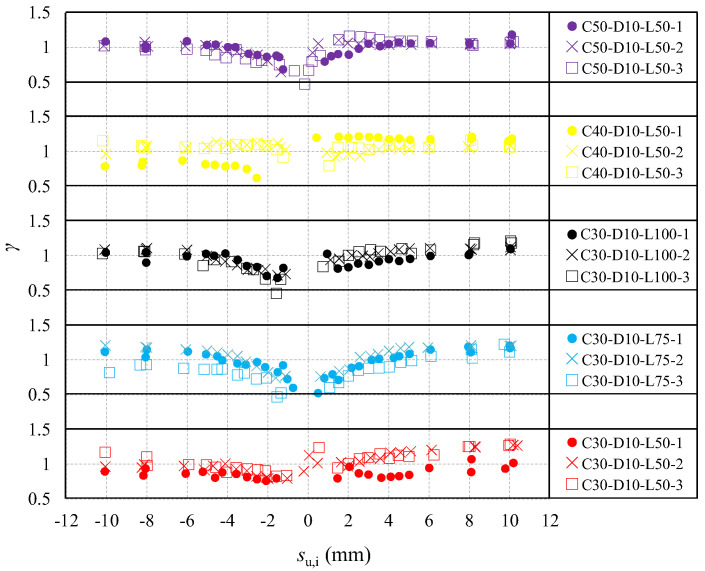
Values of *γ* in each loading cycle.

Figure 13 shows the relationship between the test results of *τ*_f_ and *τ*_u,i_, where *τ*_f_ represents $\tau_{f}^{+}$ and $-\tau_{f}^{-}$, and *τ*_u,i_ represents $-\tau_{u,i}^{-}$ and $\tau_{u,i}^{+}$. As seen from Figure 13, |*τ*_f_| increases with the increasing |*τ*_u,i_| for all specimens, and distributes around the curve calculated by Equation (29). Figure 14 shows the values of *γ* at peak slips *s*_u,i_ of each cycle, where *γ* is the ratio of the calculated result to the test result of *τ*_f_, and *s*_u,i_ represents $-s_{u,i}^{-}$ and $s_{u,i}^{+}$. As seen in Figure 14, *γ* is basically ranges between 0.75–1.25, which indicates Equation (29) could predict $\tau_{f}^{+}$ and $\tau_{f}^{-}$ with an acceptable level of accuracy.

#### 4.3.2. Bond Stresses $\tau_{n}^{+}$ and $\tau_{n}^{-}$

**Figure 15:**
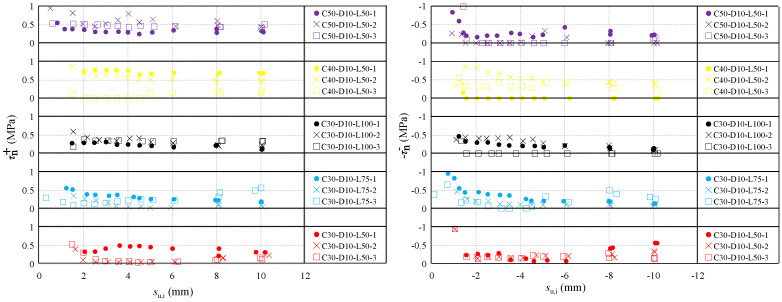
Bond stresses $\tau_{n}^{+}$ and $-\tau_{n}^{-}$ in each loading cycle.

The bond stresses $\tau_{n}^{+}$ and $\tau_{n}^{-}$ are the lowest for the IJK and MNG_1_ segments for each loading cycle, respectively. Figure 15 presents the test results of $\tau_{n}^{+}$ and $\tau_{n}^{-}$ in each loading cycle for all specimens. As seen in Figure 15, both $\tau_{n}^{+}$ and $\tau_{n}^{-}$ change slightly in different loading cycles for the specimens with different kinds of concrete compression strength and embedment length, which range between 0–1 Mpa. Therefore, the calculated values of $\tau_{n}^{+}$ and $\tau_{n}^{-}$ in the model could adopt the average values of test results, which are $\tau_{n}^{+}=0.343$ MPa and $-\tau_{n}^{-}=-0.236$ MPa.

#### 4.3.3. Decrease and Re-Increase Branches

The shape of the decrease and re-increase branches (i.e., IJK and MNG_1_ segments) are similar to the quadratic parabolas. Additionally, the IJK segment passes through point I ($s_{u,i}^{+},-\tau_{f}^{-}$), point J ($0,-\tau_{n}^{-}$), and point K ($-s_{u,i}^{-},-\tau_{u,i}^{-}$), and the MNG_1_ segment passes through point M ($-s_{u,i}^{-},\;\tau_{f}^{+}$), point N ($0,\;\tau_{n}^{+}$), and point G_1_ ($s_{u,i+1}^{+},\;\tau_{u,i+1}^{+}$). Consequently, the expressions of the decrease and re-increase branches could be described by the following quadratic equation of one variable as:

(30) $$\left\{ \begin{array}{l} \tau =a_{1}s+b_{1}s+c_{1};\;\mathrm{for}\;\mathrm{IJK}\;\mathrm{segment}\\ \tau =a_{2}s+b_{2}s+c_{2};{\;\mathrm{for}\;\mathrm{MNG}}_{1}\;\mathrm{segment} \end{array}\right.$$

where $\left\{ \begin{array}{l} a_{1}=\frac{\left(\tau_{n}^{-}-\tau_{f}^{-}\right)s_{u,i}^{-}+\left(\tau_{n}^{-}-\tau_{u,i}^{-}\right)s_{u,i}^{+}}{s_{u,i}^{+}s_{u,i}^{-}\left(s_{u,i}^{+}+s_{u,i}^{-}\right)}\\ b_{1}=\frac{\left(\tau_{n}^{-}-\tau_{f}^{-}\right)s_{u,i}^{-}{}^{2}-\left(\tau_{n}^{-}-\tau_{u,i}^{-}\right)s_{u,i}^{+}{}^{2}}{s_{u,i}^{+}s_{u,i}^{-}\left(s_{u,i}^{+}+s_{u,i}^{-}\right)}\\ c_{1}=-\tau_{n}^{-} \end{array}\right.$ and $\left\{ \begin{array}{l} a_{2}=\frac{\left(\tau_{f}^{+}-\tau_{n}^{+}\right)s_{u,i+1}^{+}+\left(\tau_{u,i}^{+}-\tau_{n}^{+}\right)s_{u,i}^{-}}{s_{u,i+1}^{+}s_{u,i}^{-}\left(s_{u,i+1}^{+}+s_{u,i}^{-}\right)}\\ b_{2}=\frac{\left(\tau_{u,i}^{+}-\tau_{n}^{+}\right)s_{u,i}^{-}{}^{2}-\left(\tau_{f}^{+}-\tau_{n}^{+}\right)s_{u,i+1}^{+}{}^{2}}{s_{u,i+1}^{+}s_{u,i}^{-}\left(s_{u,i+1}^{+}+s_{u,i}^{-}\right)}\\ c_{2}=\tau_{n}^{+} \end{array}\right.$.

Based on Equations (2)–(30), all characteristic points and segments under monotonic and reversed cyclic loading could be determined.

### 4.4. Verification of the Proposed Model

**Figure 16:**
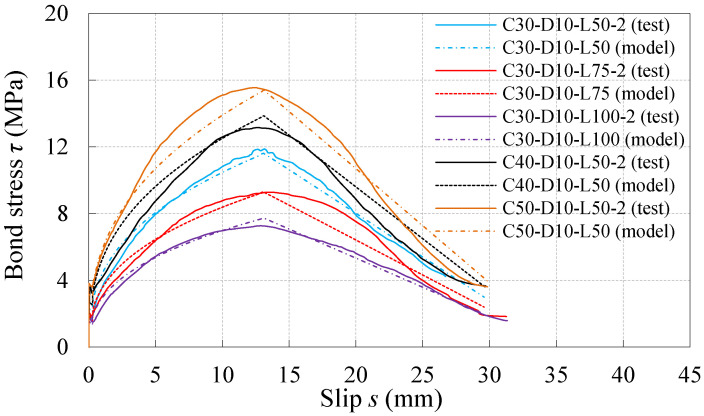
Comparison between proposed model and test curve under monotonic loading.

**Figure 17:**
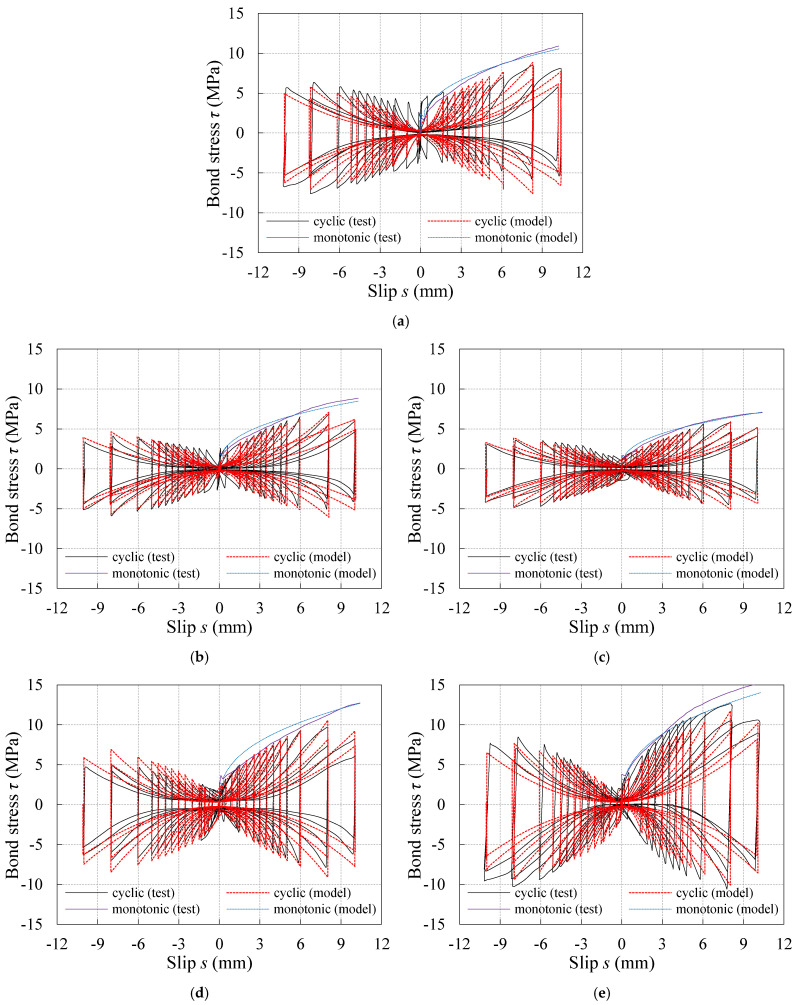
Comparison between proposed model and test curve under reversed cyclic loading: (**a**) C30-D10-L50; (**b**) C30-D10-L75; (**c**) C30-D10-L100; (**d**) C40-D10-L50; (**e**) C50-D10-L50.

To verify the proposed model, the calculated results and test results of the *τ*–*s* curves under both monotonic and reversed cyclic loading were compared for the specimens with different kinds of concrete compression strength and embedment length. One monotonic specimen and one reversed cyclic specimen were selected from each specimen group, whose maximum bond stresses were closest to the average value of three samples in the same group. By calculating each characteristic point and each segment with the loading schemes ruled in the study, the tested and calculated *τ*–*s* curves were plotted in Figure 16 and Figure 17, respectively. It can be learnt from the comparisons between the calculated curves and test curves shown in Figure 16 and Figure 17 that, although there were some discrepancies, the proposed model could predict the test results well.

## 5. Conclusions

A comprehensive experimental investigation was carried out on the bond performance of plain bars in concrete under monotonic and reversed cyclic loading. Different kinds of concrete compression strength and embedment length were considered. According to the test results and the elasticity, the *τ*–*s* model was proposed. The following conclusions can be made:

1. The bond stress–slip curves under monotonic loading present as an attenuated sine curve; the bond stress–slip curves under reversed cyclic loading present as the butterfly shape and are roughly symmetrical about the origin;
2. The maximum bond stress of plain bar in concrete decreases with the increase in embedment length, and increases with the increase in concrete compression strength, whether the specimens are subjected to monotonic loading or reversed cyclic loading.
3. The maximum bond stress and corresponding slip of plain bar in concrete under reversed cyclic loading are lower than that under monotonic loading for the specimens with the same concrete compression strength and embedment length, which illustrates that the reversed cyclic loading leads to the degeneration of bond behavior;
4. The analytical solutions for the bond stresses at the characteristic points were derived based on the elasticity, which could reflect the influence of the laws of concrete compression strength and embedment length;
5. The bond stress–slip model was proposed, which was suitable for predicting the bond stress–slip curves of plain bars in concrete under both monotonic loading and reversed cyclic loading.

## Figures and Tables

**Table 1:** Mix proportions of concrete.

Grade	Water (kg/m^3^)	Cement (kg/m^3^)	Sand (kg/m^3^)	Gravel (kg/m^3^)	Superplasticizer (kg/m^3^)	*f*_cu_ (MPa)	SD (MPa)
C30	215	347	699	1139	-	36.2	0.668
C40	215	448	591	1146	-	50.0	0.648
C50	148	375	760	1115	2.6	60.5	0.510

Note: SD represents standard deviation.

**Table 2:** Tested parameters of each specimen group.

Specimen Groups	Concrete Grade	*d* (mm)	*l* (mm)	*f*_cu_ (MPa)	Number
C30-D10-L50	C30	10	50	36.2	6
C30-D10-L75	C30	10	75	36.2	6
C30-D10-L100	C30	10	100	36.2	6
C40-D10-L50	C40	10	50	50.0	6
C50-D10-L50	C50	10	50	60.5	6

Notes: *d* represents the diameter of plain bar; *l* represents the embedment length. The named codes are illustrated as follows: “C30, C40, and C50” represent the concrete grade are C30, C40, and C50, respectively; “D10” represents the diameter of plain bar at 10 mm; “L50, L75, and L100” represent embedment lengths 50 mm, 75 mm, and 100 mm, respectively.

**Table 3:** Test results of primary parameters in bond stress–slip curves weak.

Specimens	$\tau_{e}^{m}$ (MPa)		$\tau_{a}^{m}$ (MPa)	$s_{a}^{m}$ (MPa)	$\tau_{u}^{m}$ (MPa)		$s_{u}^{m}$ (mm)	$\tau_{r}^{m}$ (MPa)	$s_{r}^{m}$ (mm)	$\tau_{u}^{+}$ (MPa)		$\tau_{u}^{-}$ (MPa)		*φ*	*ζ*	$\tau_{10,1}^{+}$ (MPa)	$\tau_{10,1}^{-}$ (MPa)	*α* ^m^	*α* ^+^	*α* ^−^
	Test	Mean			Test	Mean				Test	Mean	Test	Mean							
C30-D10-L50-1	2.11	2.20	1.85	0.23	11.32	11.87	13.34	3.12	27.79	7.76	8.50	5.88	6.85	0.76	0.72	6.91	5.48	0.457	0.653	0.416
C30-D10-L50-2	1.94		1.73	0.23	11.87		13.15	4.22	26.73	8.47		7.61		0.90		8.10	6.74	0.412	0.410	0.325
C30-D10-L50-3	2.56		2.24	0.26	12.41		12.57	2.94	27.91	9.28		7.06		0.76		6.91	6.43	0.315	0.464	0.322
C30-D10-L75-1	1.79	1.77	1.53	0.36	9.17	9.35	13.30	2.87	30.61	6.72	6.89	5.12	5.59	0.76	0.74	5.51	4.11	0.391	0.399	0.318
C30-D10-L75-2	2.03		1.85	0.20	9.28		13.29	1.83	31.27	6.94		5.89		0.85		5.95	5.14	0.363	0.389	0.343
C30-D10-L75-3	1.49		1.22	0.32	9.61		13.09	2.52	28.84	7.02		5.78		0.82		6.25	4.98	0.375	0.569	0.390
C30-D10-L100-1	1.38	1.51	1.13	0.30	6.83	7.23	13.42	1.41	29.76	5.61	5.84	4.47	4.85	0.80	0.81	4.83	3.71	0.317	0.456	0.384
C30-D10-L100-2	1.69		1.49	0.30	7.27		12.88	1.58	31.33	5.80		4.85		0.84		5.15	4.22	0.346	0.433	0.373
C30-D10-L100-3	1.48		1.31	0.28	7.60		13.44	2.04	30.79	6.11		5.24		0.86		6.00	4.94	0.359	0.539	0.579
C40-D10-L50-1	3.01	2.84	2.68	0.20	12.79	13.24	12.96	2.70	30.14	9.29	9.81	7.06	7.28	0.76	0.74	7.80	6.07	0.382	0.421	0.478
C40-D10-L50-2	3.58		3.23	0.25	13.16		12.68	3.67	29.72	9.73		7.04		0.72		8.25	6.40	0.414	0.401	0.349
C40-D10-L50-3	1.94		1.64	0.36	13.76		13.11	4.30	30.06	10.42		7.75		0.74		9.52	6.97	0.388	0.466	0.350
C50-D10-L50-1	2.75	3.20	2.52	0.28	14.87	15.38	13.39	3.47	28.79	12.14	12.52	10.41	10.12	0.86	0.81	10.57	8.55	0.359	0.445	0.446
C50-D10-L50-2	3.80		3.64	0.25	15.55		12.54	3.61	29.85	12.65		10.26		0.81		10.61	9.56	0.352	0.401	0.462
C50-D10-L50-3	3.03		2.80	0.22	15.71		13.62	4.22	30.59	12.77		9.70		0.76		11.36	8.44	0.422	0.439	0.424
Mean value				0.269			13.12		29.61					0.800	0.763			0.377	0.459	0.397
SD				0.050			0.332		1.347					0.052	0.038			0.039	0.074	0.072

**Table 4:** Test results and calculated results of characteristic points.

Specimens	$\tau_{e}^{m}$ (MPa)		Error(%)	$\tau_{u}^{m}$ (MPa)		Error(%)	$\tau_{u}^{+}$ (MPa)		Error(%)	$\tau_{u}^{-}$ (MPa)		Error(%)
	Test	Calculated		Test	Calculated		Test	Calculated		Test	Calculated	
C30-D10-L50	2.20	2.34	6.43	11.87	11.60	−2.22	8.50	8.85	4.11	6.85	7.08	3.39
C30-D10-L75	1.77	1.88	5.91	9.35	9.28	−0.75	6.89	7.08	2.73	5.59	5.67	1.27
C30-D10-L100	1.51	1.56	3.17	7.23	7.73	6.93	5.84	5.90	1.00	4.85	4.72	−2.73
C40-D10-L50	2.84	2.80	−1.53	13.24	13.86	4.70	9.81	10.57	7.74	7.28	8.46	16.18
C50-D10-L50	3.20	3.11	−2.74	15.38	15.39	0.08	12.52	11.74	−6.22	10.12	9.39	−7.21

Note: Error = (Calculated value − Test value)/Test value.

## Data Availability

The data presented in this study are available on request from the corresponding author. The data are not publicly available due to privacy.

## References

[1] Hertanto E. (2005). Seismic Assessment of pre-1970s Reinforced Concrete Structures. Ph.D. Thesis.

[2] Hu Z., Shah Y.I., Yao P. (2021). Experimental and numerical study on interface bond strength and anchorage performance of steel bars within prefabricated concrete. Materials.

[3] Tang C.-W., Cheng C.-K. (2020). Modeling local bond stress–slip relationships of reinforcing bars embedded in concrete with different strengths. Materials.

[4] Arani K.K., Marefat M.S., Amrollahi-Biucky A., Khanmohammadi M. (2010). Experimental seismic evaluation of old concrete columns reinforced by plain bars. Struct. Des. Tall Spec. Build..

[5] Xiao J., Falkner H. (2007). Bond behaviour between recycled aggregate concrete and steel rebars. Constr. Build. Mater..

[6] Hossain K.M.A. (2008). Bond characteristics of plain and deformed bars in lightweight pumice concrete. Constr. Build. Mater..

[7] Li F., Yu K., Ding X., Li C. (2013). Bond properties of plain steel bar in concrete with machine-made sand. Appl. Mech. Mater..

[8] Wu Z., Deng M., Zhang Y., Chen H., Liu J., Tian T. (2023). Bond behavior of plain bar in highly ductile fiber-reinforced concrete (HDC) subjected to monotonic and repeated loading. J. Build. Eng..

[9] Purnomo H., Chalid M., Pamudji G., Arrifian T.W. (2022). Bond–slip relationship between sand-coated polypropylene coarse aggregate concrete and plain rebar. Materials.

[10] Jiang T., Zhang X., Wu Z., Abdellahi M.M. (2017). Bond-slip response of plain bars embedded in self-compacting lightweight aggregate concrete under lateral tensions. J. Mater. Civ. Eng..

[11] Faleschini F., Santamaria A., Zanini M.A., San José J.T., Pellegrino C. (2017). Bond between steel reinforcement bars and Electric Arc Furnace slag concrete. Mater. Struct..

[12] Deng M., Pan J., Sun H. (2018). Bond behavior of steel bar embedded in Engineered Cementitious Composites under pullout load. Constr. Build. Mater..

[13] Shang H., Zhao T., Cao W. (2015). Bond behavior between steel bar and recycled aggregate concrete after freeze-thaw cycles. Cold Reg. Sci. Technol..

[14] Ma Y., Guo Z., Wang L., Zhang J. (2017). Experimental investigation of corrosion effect on bond behavior between reinforcing bar and concrete. Constr. Build. Mater..

[15] Robuschi S., Sumearll J., Fernandez I., Lundgren K. (2020). Bond of naturally corroded, plain reinforcing bars in concrete. Struct. Infrastruct. Eng..

[16] Fabbrocino G., Verderame G.M., Manfredi G. (2005). Experimental behaviour of anchored smooth rebars in old type reinforced concrete buildings. Eng. Struct..

[17] Zhang X., Dong W., Zheng J., Wu Z., Hu Y., Li Q. (2014). Bond behavior of plain round bars embedded in concrete subjected to lateral tension. Constr. Build. Mater..

[18] Wu Z., Zhang X., Zheng J., Hu Y., Li Q. (2014). Bond behavior of plain round bars embedded in concrete subjected to biaxial lateral tensile-compressive stresses. J. Struct. Eng..

[19] Xu F., Wu Z., Zheng J., Hu Y., Li Q. (2014). Bond behavior of plain round bars in concrete under complex lateral pressures. ACI Struct. J..

[20] Li X., Wu Z., Zheng J., Dong W. (2015). Effect of loading rate on the bond behavior of plain round bars in concrete under lateral pressure. Constr. Build. Mater..

[21] Li X., Wu Z., Zheng J., Cao Q. (2020). Rate-dependent bond performance of plain bars in concrete under biaxial transverse tensions. Eng. Struct..

[22] Melo J., Rossetto T., Varum H. (2015). Experimental study of bond-slip in RC structural elements with plain bars. Mater. Struct..

[23] Xing G., Zhou C., Wu T., Liu B. (2015). Experimental study on bond behavior between plain reinforcing bars and concrete. Adv. Mater. Sci. Eng..

[24] Cairns J. (2021). Local bond–slip model for plain surface reinforcement. Struct. Concr..

[25] Eligehausen R., Popov E.P., Bertero V.V. (1983). Local and Bond Stress-Slip Relationships of Deformed Bars under Generalized Excitations.

[26] Zhang X., Wu Z., Zheng J., Dong W., Bouchair A. (2016). Ultimate bond strength of plain round bars embedded in concrete subjected to uniform lateral tension. Constr. Build. Mater..

[27] Pan J., Deng M., Sun H. (2021). Bond behavior of plain round bar embedded in high ductile concrete under monotonic and cyclic loading. Struct. Concr..

[28] Verderame G.M., Ricci P., Carlo G.D., Manfredi G. (2009). Cyclic bond behaviour of plain bars. Part I: Experimental investigation. Constr. Build. Mater..

[29] Verderame G.M., Carlo G.D., Ricci P., Fabbrocino G. (2009). Cyclic bond behaviour of plain bars. Part II: Analytical investigation. Constr. Build. Mater..

[30] Xa S., Nie B., Li A. (2019). Bond properties for plain bars in frost-damaged concrete. Mag. Concr. Res..

[31] Xu S., Li A., Wang H. (2017). Bond properties for deformed steel bar in frost-damaged concrete under monotonic and reversed cyclic loading. Constr. Build. Mater..

[32] Hu X., Peng G., Niu D., Wang J. (2019). Bond properties of deformed steel bars in concrete during construction under reversed cyclic loading. Constr. Build. Mater..

[33] Shen D., Wen C., Zhu P., Li M., Ojha B., Li C. (2020). Bond behavior between basalt fiber-reinforced polymer bars and concrete under cyclic loading. Constr. Build. Mater..

[34] Zhao J., Li X., Zhang X. (2021). Experimental and theoretical research on bond performance between CFRP bar and concrete under monotonic and reversed cyclic loading. Eng. Struct..

[35] Cai G. (2014). Seismic Performance and Evaluation of Resilient Circular Concrete Columns. Ph.D. Thesis.

[36] Sargsyan G., Cai G., Takeuchi T., Sun Y. Seismic behaviour and assessment of drift-hardening concrete columns. Proceedings of the 16th World Conference on Earthquake (16WCEE).

[37] Grigor S. (2017). Cyclic Behavior and Evaluation of Drift-Hardening Concrete Columns. Ph.D. Thesis.

[38] Liu Z., Zhao H., Sun Y., Han R., Zhao Q. (2020). Seismic performance of circular concrete columns reinforced by PC Strands. J. Adv. Concr. Technol..

[39] (2011). Specification for Mix Proportion Design of Ordinary Concrete.

[40] (2019). Standard for Test Methods of Concrete Physical and Mechanical Properties.

[41] (2021). Metallic Materials-Tensile Testing—Part 1: Method of Test at Room Temperature.

[42] (2014). Standard Test Method for Bond Strength of Fiber Reinforced Polymer Matrix Composite Bars to Concrete by Pull-Out Testing.

[43] Rabinowicz E. (1995). Friction and Wear of Materials.

[44] Zhao W., Xiao J. (2011). On bond-slip constitutive model between ribbed steel bars and concrete. Eng. Mech..

[45] Timoshenko S.P., Goodier J.N. (1982). Theory of Elasticity.

[46] (2015). Code for Design of Concrete Structure.

[47] Feldman L.R., Bartlett F.M. (2007). Bond stress along plain steel reinforcing bars in pullout specimens. ACI Struct. J..

